# High-throughput SNP discovery and assay development in common bean

**DOI:** 10.1186/1471-2164-11-475

**Published:** 2010-08-16

**Authors:** David L Hyten, Qijian Song, Edward W Fickus, Charles V Quigley, Jong-Sung Lim, Ik-Young Choi, Eun-Young Hwang, Marcial Pastor-Corrales, Perry B Cregan

**Affiliations:** 1 Soybean Genomics and Improvement Laboratory, U.S. Department of Agriculture, Agricultural Research Service, Beltsville, Maryland 20705, USA; 2 Department Plant Science and Landscape Architecture, University of Maryland, College Park, MD 20742, USA; 3 Genome Research Laboratory/National Instrumentation Center for Environmental Management, Seoul National University, Seoul 151-921, South Korea

## Abstract

**Background:**

Next generation sequencing has significantly increased the speed at which single nucleotide polymorphisms (SNPs) can be discovered and subsequently used as molecular markers for research. Unfortunately, for species such as common bean (*Phaseolus vulgaris *L.) which do not have a whole genome sequence available, the use of next generation sequencing for SNP discovery is much more difficult and costly. To this end we developed a method which couples sequences obtained from the Roche 454-FLX system (454) with the Illumina Genome Analyzer (GA) for high-throughput SNP discovery.

**Results:**

Using a multi-tier reduced representation library we discovered a total of 3,487 SNPs of which 2,795 contained sufficient flanking genomic sequence for SNP assay development. Using Sanger sequencing to determine the validation rate of these SNPs, we found that 86% are likely to be true SNPs. Furthermore, we designed a GoldenGate assay which contained 1,050 of the 3,487 predicted SNPs. A total of 827 of the 1,050 SNPs produced a working GoldenGate assay (79%).

**Conclusions:**

Through combining two next generation sequencing techniques we have developed a method that allows high-throughput SNP discovery in any diploid organism without the need of a whole genome sequence or the creation of normalized cDNA libraries. The need to only perform one 454 run and one GA sequencer run allows high-throughput SNP discovery with sufficient sequence for assay development to be performed in organisms, such as common bean, which have limited genomic resources.

## Background

DNA markers are invaluable tools across many species for use in QTL mapping, marker assisted selection, association analysis, and fine mapping for cloning of genes of interest. By far the most abundant source of DNA variation for marker development is the single nucleotide polymorphism (SNP). The SNP marker has become the marker of choice for many research applications because of the abundance of SNPs and the several technologies available for the high-multiplex assay of SNPs [1]. The existence of high-throughput methods for assaying SNPs is continually reducing the cost of genotyping and is making these high-throughput methods accessible to more researchers. However, the cost of SNP discovery still remains relatively high, especially for organisms that do not have a sequenced genome. Thus, this cheaper high-throughput genotyping technology is unavailable to many researchers.

SNP discovery in most species has generally relied upon *in silico *analysis of existing sequence data or the resequencing of a small number of genotypes for the identification of sequence variants in existing sequence data [2-5]. While these methods of resequencing have been successful for SNP discovery, they are time consuming and expensive. Recently, this has changed due to the availability of high-throughput sequencing technologies.

For complex animal and plant genomes such as cattle and soybean, high-throughput SNP discovery has been demonstrated using the next generation sequencing on the Genome Analyzer (GA) platform from Illumina, Inc. (subsequently referred to as GA sequencing) [6,7]. In both cattle and soybean, GA sequencing was performed on reduced representation libraries (RRL) which reduced the complexity of the portion of the genome that was sequenced after a restriction digestion of the DNA and a size selection of a proportion of the resulting DNA fragments [6,7]. In cattle this approach successfully identified 62,042 putative SNPs shown to have a 91% validation rate [7]. In soybean 7,108 to 25,047 SNPs were predicted with a validation rate ranging from 79% to 92.5% [6]. While the use of next-generation sequencing to sequence RRL appears to be very efficient for SNP discovery, a whole genome sequence for the alignment is still required because of the short read lengths produced by GA sequencing.

Many animal and plant species do not have whole genome sequences available; thus, scientists working with these species have not been able to fully take advantage of next generation sequencing for SNP discovery. One such species is common bean (*Phaseolus vulgaris *L., Fabaceae), a predominantly self-pollinating crop of world-wide importance for its nutritional value. Because relatively limited resources have been devoted to marker development in common bean, there are currently few SNP markers available [8,9] for genetic improvement. Our objective was to create a multi-tier reduced representation library (mtRRL) through a series of restriction digestions and gel size selection followed by high-throughput DNA sequencing for the discovery of large numbers of SNPs in common bean with sufficient flanking sequence for GoldenGate assay design.

## Results

**Figure 1:**
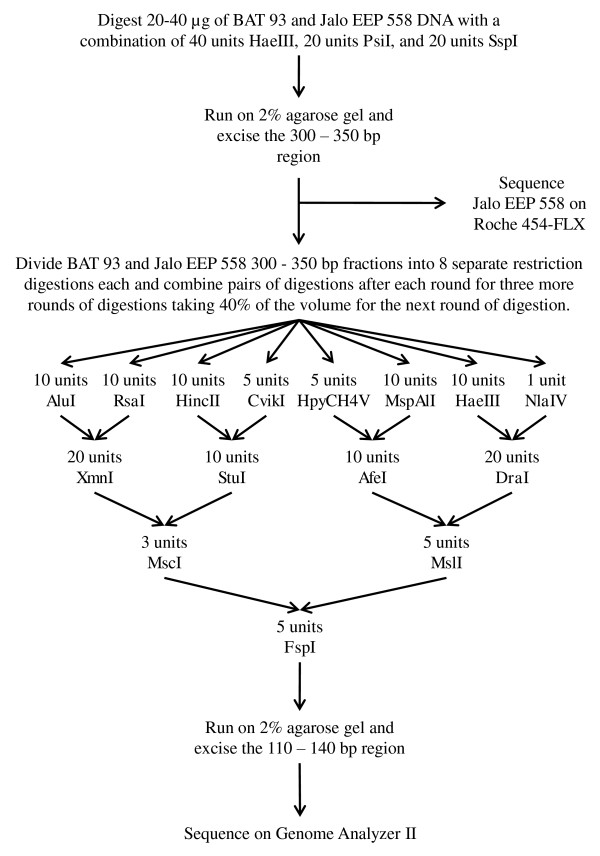
**Sequencing of the multi-tier reduced representation library**. Diagram of the series of restriction digestions used to create and sequence the multitier reduced representation library.

To accomplish SNP discovery using only sequence produced by next generation sequence analysis, mtRRLs were created of the common bean genotypes Jalo EEP 558 and BAT 93. The first tier restriction consisted of digesting Jalo EEP 558 DNA with three restriction enzymes followed by gel size selection of the 300 to 350 bp DNA fragments. The sequencing of the first tier size selected DNA was performed using the Roche 454-FLX sequencing method [10] (subsequently referred to as 454 sequencing) to produce the genomic reference sequence. This genomic reference sequence would be used to align GA sequencing reads produced by sequencing the 110 to 140 bp gel size selected DNA fragments. The 110 to 140 bp size selected DNA fragments were produced from a series of restriction digestions performed on the 300 to 350 bp DNA fragments, first tier restriction, of both the Jalo EEP 558 and BAT 93 genotypes (Figure 1). The discovery of SNPs that occur toward the middle of the 454 reference sequence would then have sufficient flanking sequence for GoldenGate assay design.

### 454 Sequencing

A total of 576,264 reads were obtained from one run of the 454 sequencer to yield 139 Mbp of DNA sequence of the cultivar Jalo EEP 558. The sequence was assembled into 160,036 reference sequences, including 67,340 contigs and 92,696 singleton sequences. A total of 107 contigs/singletons (33,688 bp) that aligned with chloroplast or mitochondrial DNA were eliminated as were 2,432 contigs/singletons (269,338 bases) which were less than 61 bases or with "N" for more than 75% of their total length leaving 99.9% of the reads with an average quality score greater than 20. This resulted in 157,497 contigs/singletons with a total length of 36 Mbp, an average length of 230 bp per sequence read and a median length of 241.

### Illumina GA Sequencing and alignment to the 454 sequences

A total of 1,010 Mbp of BAT 93 and 1,608 Mbp of Jalo EEP 558 DNA sequence was obtained from the GA sequencing. The length of individual reads ranged from 36 bp to 42 bp. The sequences were aligned with the 454 reference sequences using the software program ELAND.

### SNP Discovery and Validation

A total of 16,082,341 reads or 647 Mbp BAT 93 GA sequences were aligned to Jalo EEP 558, 454 sequencer consensus and singleton reads. This alignment identified 35,784 SNPs with minimum quality score of 10 and read depth of 3 using the CASAVA software. No insertion/deletions (INDELs) were called since a common sequencing error of the 454-FLX system is miscalling the number of bases in homopolymers [10] and the GoldenGate assay is not able to assay INDELs.

By mapping and assembling the Jalo EEP 558 reference sequences with Jalo EEP 558 short reads, SNPs from homologous or paralogous regions could be identified. A total of 1,307 SNPs from such regions were eliminated from the 35,784 SNPs that were initially identified.

For the remaining 34,477 candidate SNPs, the SNP allele in the Jalo EEP 558 reference sequence was replaced with the BAT 93 allele and the Jalo EEP 558 GA short reads were aligned to the BAT 93 allele consensus sequence. This step resulted in confirming a total of 5,165 SNPs of the 34,477 candidate SNPs.

The SNP number was further reduced to 4,341 after filtering the SNPs residing in the fragments which were significantly aligned with repetitive sequences http://phaseolus.genomics.purdue.edu/data/pv_gba_recon_repeats.fasta (269 SNPs). Since the roots that were used for DNA extraction were collected from unsterilized soil, the remaining SNPs were also screened for bacterial sequences from GenBank which eliminated an additional 555 SNPs.

A total of 192 primer pairs were designed to 192 randomly chosen SNPs out of the 4,341 candidate SNPs. A total of 108 primer pairs produced a good robust sequence tagged site with high quality sequence surrounding the candidate SNP. Of the 108 candidate SNPs, 93 (86.1%) contained the predicted SNP.

### GoldenGate SNP Validation

Before the 4,341 SNPs were submitted to Illumina for GoldenGate assay design they were further screened to ensure that all BAT 93 GA sequences only contained one allele for each candidate SNP. Of the 4,341 SNPs called by CASAVA using three or more GA sequences, there was 854 SNPs that were eliminated because at least one GA sequence had a different allele than the called BAT 93 consensus base. The remaining 3,487 SNPs [Additional file 1] were submitted for scoring by the Illumina GoldenGate assay design tool. Of the 3,487 SNPs, 2,795 had a SNP score ≥ 0.4 which Illumina uses to predict a moderate rate of success for obtaining a successful GoldenGate assay of which 2,255 had a SNP score ≥ 0.6 which Illumina uses to predict a high rate of success for converting a SNP into a working GoldenGate assay.

To design a 1,536 GoldenGate assay, 1,050 candidate SNPs were chosen which had a SNP score ≥ 0.843 and an average SNP score of 0.93. The remaining 486 SNPs were SNPs discovered through Sanger resequencing and were not part of the validation for this project (data not shown). The 1,536 GoldenGate assay named PvOPA-1 (***P**haseolus **v**ulgaris ***o**ligo **p**ool **a**ll -1) produced 827 successful GoldenGate assays out of the 1,050 candidate SNPs. A total of 822 of these successful GoldenGate assays were found to be polymorphic between BAT 93 and Jalo EEP 558. While the five other successful GoldenGate assays were polymorphic in a set of 96 diverse common bean germplasm accessions. These five additional polymorphic markers were likely not polymorphic between BAT 93 and Jalo EEP 558 due to residual heterogeneity in these two lines and the DNA used for the sequencing was a separate extraction from the DNA that was used for the GoldenGate analysis.

## Discussion

The multi-tier reduced representation library successfully took advantage of the strengths of two next generation sequencing methods. The main advantage of the 454-FLX system is the generation of longer reads than the GA system. Maughan et al., [11] were able to sequence a reduced representation library using only the 454-FLX system for SNP discovery in Amaranth in which they estimated that their RRL represented 10 Mbp of the 466 Mbp genome. The sequencing of the first tier in the common bean RRL produced 67,340 contigs and 92,696 singleton sequences. After elimination of chloroplast and mitochondrial DNA a total of 36 Mbp of unique sequence was obtained. The high number of singleton sequences and the lack of read-depth in the contigs likely indicated that this 36 Mbp did not include all fragments that were in the 300 to 350 bp size range. Since our reduced representation library likely contains a larger proportion of the estimated 600 Mb common bean genome [12] than Maughan et al., [11] isolated from Amaranth an additional sequence run of the 454-FLX system on a second genotype was unlikely to be sufficient for SNP discovery in common bean. The isolation of a larger proportion of the genome was expected since three restriction enzymes were used in the first restriction digestion instead of a single enzyme as has been used in previous studies [7,11].

The advantage of the GA system is that it produces millions more reads than the 454-FLX system at a much lower cost but these reads are considerably shorter. While sequencing the first tier with 454-FLX system alone was inefficient for SNP discovery in common bean, it did produce 300 to 350 bp sequences which we were able to utilize as a reference sequence to align the shorter, but much more numerous, GA sequences. The further reduction of the 300 to 350 bp first-tier fraction to 110 to 140 bp fragments allowed the end sequencing of those fragments with the GA system. This smaller second-tier fraction derived from the 300 to 350 bp fragments ensured that many of the GA reads occurred at various positions within the 454-FLX fragments giving ample flanking sequence on either side of the predicted SNP.

This process predicted SNPs at an 86% success rate when GA reads of both BAT 93 and Jalo EEP 558 were used to predict the SNP. This is similar to the 92.5% obtained in soybean using two or more reads to predict a SNP [6] and 91% obtained in cattle when using two or more reads to predict a SNP [7] when sequencing a reduced representation library with GA sequencing aligned to a reference genome. Barbazuk et al., [13] obtained an 85% validation rate when using a 454 GS-20 run to sequence the transcriptome of two inbred maize lines when there was no reference genome sequence available. The 86% success rate would likely be increased with additional sequencing which could help identify paralogous sequence variants and would help eliminate SNPs called due to sequencing errors. Longer sequencing reads that can now be obtained with the GAIIx or the Illumina HiSeq 2000 should also allow for better identification of paralogous sequence causing a false positive SNP call. Even with the longer reads of 100 bp available for the GAIIx or HiSeq 2000 it is likely that a reference sequence of at least 200 bp in the form of a 454 sequence would still be needed to obtain enough context sequence surrounding the SNP to have a high probability of converting that SNP into a usable assay. Once the whole genome sequence of common bean is available, a reanalysis of the data should also increase the success rate of SNP prediction.

While using both BAT 93 and Jalo EEP 558 GA sequences gave a high validation rate, requiring a Jalo EEP 558 GA read to validate a SNP considerably reduced the final number of predicted SNPs. However, this step was necessary in order to eliminate paralogous sequence variants. This large decrease in predicted SNPs also indicated that with the mtRRL constructed here, many more SNPs could potentially be confirmed with additional Jalo EEP 558 sequencing. Even without additional sequence runs we were able to design 1050 GoldenGate assays from the sequence data produced from the 454-FLX system and obtained working GoldenGate assays for 79% of the predicted SNPs. It has been shown in soybean that the conversion of confirmed SNPs into working GoldenGate assays is approximately 90% [14]. Using unconfirmed SNPs as discovered in this study the percent of working GoldenGate assays should be the product of the validation rate by the conversion rate of confirmed SNPs. If the 86% validation rate obtained by Sanger sequencing is used than obtaining a 79% rate of working GoldenGate assays would suggest that for common bean the GoldenGate assay had a 92% conversion rate for real SNPs which is slightly higher than what has been obtained with soybean.

All the SNP resources developed in the present study have not been exploited in the GoldenGate assay: 1,205 SNPs are still available with predicted success rates equal to the 1,050 SNPs used for the GoldenGate assay development. In addition there are another 540 SNPs with predicted success rates that should be slightly lower than the 79% conversion rate that we obtained that could still be developed into assays. Each of the 3,487 SNPs has sufficient flanking sequence that a variety of other SNP detection methods could be used in place of the GoldenGate assay [15].

It is expected that the 3,487 SNPs should randomly distribute throughout the genome since the enzymes chosen were not chosen to enrich for genic sequence. Because of this random distribution, it is expected that when these SNPs are genetically mapped they will cluster depending upon the amount of heterochromatic DNA present in common bean. It has been shown in the closely related legume soybean, that 57% of the genome is heterochromatic DNA and that recombination is severely suppressed [16]. It has been estimated that in common bean approximately 48% of the genome that is heterochromatic [17]. While this predicts that half of the SNPs discovered will genetically cluster, they will still be useful in assembling the genome sequence of common bean [6] which is currently in progress (Scott Jackson, personal communication). It is interesting to note that in soybean, 21.6% of high-confidence genes are located in the heterochromatic DNA [16] and that a SNP discovery method using the transcriptome would likely demonstrate some clustering in the heterochromatic DNA. This can be observed in the recent SNP discovery project in barley which only used cDNA for SNP discovery and still demonstrates some clustering around the pericentromeric regions on the barley chromosomes which are likely to be heterochromatic [18].

Other methods that have used next generation sequencing for SNP discovery in organisms without a whole genome sequence reduced the complexity of the genome through the creation of normalized cDNA libraries or through capture arrays that were then sequenced using a 454-FLX system [19-21]. While these methods can be very successful for SNP discovery, they still require the creation of normalized cDNA libraries or a capture array which can be expensive and time consuming. Another drawback with SNP discovery using the transcriptome is that genes are more conserved than non-coding DNA which will lead to the discovery of fewer SNPs [5]. The more conserved sequence will also lead to primers or probes hybridizing to both the gene sequence that contains the SNP as well as any conserved paralogous sequence, thereby decreasing the success rate of assays for such SNP [22]. In addition, without a genomic reference sequence, the proportion of successful SNP assays designed to cDNA sequence will also be decreased due to the present of introns interfering with oligo hybridization.

## Conclusions

Coupling two next generation sequencing methods with a multi-tier reduced representation library allowed for high-throughput SNP discovery in common bean, an organism for which no whole genome sequence is available and without the need to create normalized cDNA libraries. In total, one run of the 454-FLX and one run of the GA sequencer were sufficient to discover 4,341 SNPs for a total cost under $15,000. Since this study was initially conducted, the read lengths and number of reads have increased significantly for both the 454-FLX and the GA sequencers, thereby allowing a larger number of SNPs to be discovered for a similar cost. This total cost makes the discovery of SNPs attainable for many researchers working with organisms for which limited funding is available. The utility of this SNP discovery method is also demonstrated by the amount of flanking genomic sequence around the SNP which is sufficient to generate assays to covert these SNPs into usable molecular markers for genetic research.

## Methods

### Reduced Representation Library Construction

DNA of BAT 93 and Jalo EEP 558 were isolated from bulk root tissue of 100-150 plants as described by Keim et al., [23]. The first RRL created for 454-FLX sequencing was developed by digesting a total of 20 to 40 μg of Jalo EEP 558 DNA with 40 units of *Hae*III, 20 units of *Psi*I, and 20 units of *Ssp*I (New England Biolabs, Ipswich, MA) in a total volume of 210 μl. The restriction enzymes were chosen because the resulting digests did not produce any banding in the 300 to 350 bp range as assessed by 2% agarose gel electrophoresis. Such banding would be indicative of restriction sites in highly repetitive elements. The restriction digestion was carried out overnight at 37°. The digested DNA was then run on a 2% agarose gel and the digestion products were excised from the gel in the 300 to 350 bp size range. The QIAquick Gel Extraction Kit (Qiagen, Hilden, Germany) was used as per the manufacturer's protocol to obtain the size selected DNA. A total of 1,000 ng of the 300 to 350 bp fragments were sequenced using the 454-FLX Life Sciences sequencer (Roche Applied Sciences, Indianapolis, IN, USA) as per the manufacturer's instructions.

The same restriction digestion and size selection was also performed on BAT 93 DNA to obtain a digestion product ranging from 300 to 350 bp. The 300 to 350 bp RLL of both Jalo EEP 558 and BAT 93 DNA were put through a series of restriction digestions as shown in Figure 1. The restriction enzymes were chosen because the resulting digests did not produce any banding in the 110 to 140 bp range as assessed by 2% agarose gel electrophoresis. The resulting DNA was run on a 2% agarose gel and the digestion products ranging from 110 to 140 bp were excised from the gel. The resulting second-tier RLL DNA was then sequenced on the Genome Analyzer II (Illumina, Inc; San Diego, CA, USA) as per the manufacturer's protocol. All sequence data produced from the 454-FLX and GA sequencing have been deposited in the NCBI, Sequence Read Archive [GenBank: SRA020162-SRA020164].

### SNP discovery and Validation

The sequences of Jalo EEP 558 from the Roche 454-FLX system were assembled using the software 454 GS *de novo *assembler, Newbler from Roche Applied Science to generate the Jalo EEP 558 reference sequence. The default overlap detection parameters with the assembler were used for assembly. Each base from the contigs and the singletons was screened for its Phred quality. If the quality was less than 20, the corresponding base was changed to "N". The contigs and singletons less than 61 bases or with an "N" at more than 75% of their total length were eliminated. The sequences were then Blasted to the sequences of *Phaseolus vulgaris *chloroplast [GenBank:NC_009259] using standalone megablast with W = 30. Any sequences that aligned to the *P. vulgaris *chloroplast DNA were eliminated.

The short reads of BAT 93 and Jalo EEP 558 generated by the Illumina Genome Analyzer II were analyzed and called by the Sequence Analysis Software of Pipeline version 1.4 of the Genome Analyzer (Illumina, Inc., San Diego, CA). ELAND of the Pipeline version 1.4 was used for the alignment of the BAT 93 short reads to the Jalo EEP 558 reference sequences. A SNP allele was called using the software package of the Consensus Assessment of Sequence and Variation (CASAVA) (Illumina, Inc., San Diego, CA). The constraints of minimum allele call score of 10 at the SNP position and minimum read depth of 3 were imposed to filter SNPs. To reduce the SNPs associated with the homologs or paralogs, the Jalo EEP 558 reference sequences were further mapped and assembled with the short reads of Jalo EEP 558 sequences by CASAVA, the SNPs which were identical to the SNPs kept in the previous procedure were eliminated. In addition, the Jalo EEP 558 reference sequence at the positions where SNPs were called was replaced with the BAT 93 alleles and the Jalo EEP 558 GA short reads were aligned to the BAT 93 allele consensus sequence. SNPs were called and used for the verification of the SNPs called from BAT 93 short reads vs. Jalo EEP 558 reference sequences.

The SNPs residing in the fragments which were significantly aligned with common bean repetitive sequences http://phaseolus.genomics.purdue.edu/data/pv_gba_recon_repeats.fasta or with bacterial sequences deposited in GenBank, as identified with the Megablast software with W = 30, were also eliminated. The screening against bacterial sequences was performed at the end of the SNP discovery pipeline rather than at the beginning due to the size of the data set at the beginning of the SNP pipeline which would have required significantly more computational time.

Based upon the underlying Jalo EEP 558 454-FLX reference sequence, polymerase chain reaction (PCR) primers were designed to the flanking sequence of a random set of 192 putative SNPs using Primer3 [24]. The targeted PCR product lengths ranged from 80 to 300 bases, with an annealing temperature of 58°C. In order to reduce the likelihood of non-specific annealing of primers to the reference sequences, the primer sets were examined with e-PCR software [25] with the parameter of N = 3 and G = 1. Primer sets that were aligned to more than one reference sequence were discarded. Initial amplification, sequence analysis and alignment for validation of putative SNPs between BAT 93 and Jalo EEP 558 were performed as described by Choi et al. [22]. The above procedures were completed using Perl script, AWK script and SAS procedures, such as SQL, SURVEYSELECT etc. [26].

### GoldenGate assay

The 4,341 predicted SNPs were screened to insure that all GA sequence reads that predicted a particular SNP contained the same allele. After this screen the remaining 3,487 SNPs were submitted to Illumina Inc. to obtain a SNP score by the Illumina assay design tool for the GoldenGate assay. A SNP score > 0.6 is used by Illumina to predict those SNPs which will have high conversion rates into successful GoldenGate assays [27] and has been shown to predict a 91% conversion rate in soybean [14].

The 1,536 GoldenGate assay contained a total of 1,050 candidate SNPs from the mtRRL method described here, all of which had a SNP score ≥ 0.8. The average SNP score of the 1,050 SNPs was 0.93. The remaining 486 SNPs were discovered through other methods and their results are not reported here. The 1,536 GoldenGate assay was named PvOPA-1 (***P**haseolus **v**ulgaris ***o**ligo **p**ool **a**ll -1). The GoldenGate assay was performed on BAT 93 and Jalo EEP 558 and 96 diverse common bean germplasm accessions (Additional File 2) as per the manufacturers protocol [27,28]. The DNA for the lines was extracted as described by Keim et al. [23]. The Illumina BeadStation 500G (Illumina Inc. San Diego, CA) was used to read the 96 Sentrix Array Matrix v.7a containing the products produced from the GoldenGate assay. The resulting data were clustered using BeadStudio v.3.3 and all allele calls were visually inspected and any errors in allele calling due to improper cluster identification were corrected.

## Authors' contributions

DLH, designed and oversaw the study, designed the GoldenGate OPA, performed genotyping analysis of the GoldenGate assay, and drafted the manuscript. QS, designed and performed the SNP discovery analysis and assisted in preparing the manuscript. EWF, designed and created the multi-tier reduced representation library and performed sequencing of the validation sets and assisted in preparing the manuscript. CVQ, performed GA sequencing and assisted in preparing the manuscript. J-SL and I-YC, performed 454 sequencing. E-YH, isolated DNA and assisted with the analysis of the diverse germplasm material. MPC, provided plant material. PBC, designed and oversaw the study and assisted in preparing the manuscript. All authors read and approved the final manuscript.

## Supplementary Material

Additional file 1 **SNPs used for GoldenGate design**. The 3,487 SNPs along with the flanking sequence submitted for GoldenGate assay design. Click here for file

Additional file 2 **Diverse Germplasm**. List of the 96 diverse common bean germplasm used to test the PvOPA-1 GoldenGate assay. Click here for file
